# A Study Protocol to Increase Engagement in Evidence Based Hospital and Community Based Care Using a Serious Injection Related Infections (SIRI) Checklist and Enhanced Peer for Hospitalized PWID (ShaPe)

**DOI:** 10.21203/rs.3.rs-2546488/v1

**Published:** 2023-06-07

**Authors:** Margaret Baldwin, Madison Jeziorski, Mariel Parman, Kelly Gagnon, M Alana Nichols, Davis Bradford, Kaylee Crockett, Ellen Eaton

**Affiliations:** University of Alabama at Birmingham, Heersink School of Medicine, Division of Infectious Diseases; University of Alabama at Birmingham, Heersink School of Medicine, Division of Infectious Diseases; University of Alabama at Birmingham, Heersink School of Medicine, Division of Infectious Diseases; University of Alabama at Birmingham, Heersink School of Medicine, Division of Infectious Diseases; University of Alabama at Birmingham, Heersink School of Medicine, Division of Infectious Diseases; University of Alabama at Birmingham, Heersink School of Medicine, General Internal Medicine; University of Alabama at Birmingham, Heersink School of Medicine, Family & Community Medicine; University of Alabama at Birmingham, Heersink School of Medicine, Division of Infectious Diseases

**Keywords:** Protocol, Rural, Southern, HIV, Addiction, Opioid substance treatment, PrEP, Hospitalization

## Abstract

**Background:**

With the opioid crisis, surging methamphetamine use, and healthcare disruptions due to SARS-CoV-2, serious injection related infections (SIRIs), like endocarditis, have increased significantly. Hospitalizations for SIRI provide a unique opportunity for persons who inject drugs (PWID) to engage in addiction treatment and infection prevention, yet many providers miss opportunities for evidence-based care due to busy inpatient services and lack of awareness. To improve hospital care, we developed a 5-item SIRI Checklist for providers as a standardized reminder to offer medication for opioid use disorder (MOUD), HIV and HCV screening, harm reduction counseling, and referral to community-based care. We also formalized an Intensive Peer Recovery Coach protocol to support PWID on discharge. We hypothesized that the SIRI Checklist and Intensive Peer Intervention would increase use of hospital-based services (HIV, HCV screening, MOUD) and linkage to community-based care: PrEP prescription, MOUD prescription, and related outpatient visit(s).

**Methods:**

This is a feasibility study and randomized control trial of a checklist and intensive peer intervention for hospitalized PWID with SIRI admitted to UAB Hospital. We will recruit 60 PWID who will be randomized to one of 4 groups (SIRI Checklist, SIRI Checklist + Enhanced Peer, Enhanced Peer, and Standard of Care). Results will be analyzed using a 2x2 factorial design. We will use surveys to collect data on drug use behaviors, stigma, HIV risk, and PrEP interest and awareness. Our primary outcome of feasibility will include the ability to recruit hospitalized PWID and retain them in the study to determine post-discharge clinical outcomes. Additionally, we will explore clinical outcomes using a combination of patient surveys and electronic medical record data (HIV, HCV testing, MOUD and PrEP prescriptions).

This study is approved by UAB IRB #300009134.

**Discussion:**

This feasibility study is a necessary step in designing and testing patient-centered interventions to improve public health for rural and Southern PWID. By testing low barrier interventions that are accessible and reproducible in states without access to Medicaid expansion and robust public health infrastructure, we aim to identify models of care that promote linkage and engagement in community care.

**Trial Registration:**

NCT05480956

## Background

With the opioid crisis, surging methamphetamine use, and healthcare disruptions due to SARS-CoV-2, the U.S. is facing a rising tide of infectious consequences of drug use [1, 2]. It is estimated that 3.6 million Americans inject drugs [3], and increasingly substance use is driving ER visits and hospitalizations. Serious injection related infections (SIRI), like endocarditis, have increased significantly in recent years with hospitalizations rising 60% to 300 admissions per 100,000 persons (2009–2018) [4, 5]. Hospitalized persons who inject drugs (PWID) often have comorbid HCV (80%); a smaller percentage have HIV (4%), but many are at high risk and would benefit from HIV prevention through PrEP [6]. Only one in 3 PWID with opioid use disorder are effectively linked to medications for opioid use disorder on discharge [7]. And, in the year following a SIRI admission, PWID often experience subsequent infections, readmissions, and death. Hence, hospitalization is a golden opportunity to engage PWID who often lack insurance and access to care [1, 8].

Admissions for SIRI generally last from two to four weeks, providing an opportunity to intervene on addiction and HIV risk and transition patients to community services [9, 10]. PWID could benefit from myriad services when hospitalized that they may not otherwise receive due to lack of access, stigma, and limited public health infrastructure in rural and poor states. HIV screening, linkage to HIV prevention and treatment, and engagement in addiction treatment (i.e., MOUD) are all feasible and acceptable services for hospitalized PWID. Yet, few health systems enable these services in the acute care setting. Many providers miss opportunities for counseling and evidence-based care due to competing demands, busy inpatient services, and a lack of awareness. An emerging body of literature on SIRI has highlighted innovative hospital approaches, such as Peer Recovery Coaches, persons with lived experience in addiction in the hospital setting [11]. However, few studies have incorporated rural and Southern people to ensure delivery of services are feasible.

The use of a “checklist” can improve provider awareness, best practice, and clinical outcomes for seriously ill patients [12, 13]. Checklists are low cost and low technology reminders to providers to ensure they deliver evidence-based services to a target population. Together with a team of physicians, social workers, and peer recovery coaches, we developed a SIRI Checklist to act as a standar[14]dized reminder to offer these patients screening for HIV and HCV, medication for opioid use disorder (MOUD), consider HIV pre-exposure prophylaxis (PrEP) and referral to appropriate community-based care. Our team also formalized an Intensive Peer Intervention whereby PWID would be contacted weekly in the month following discharge to provide support, adherence reminders, and serve as a resource related to community addiction services.

We hypothesize that it is feasible to provide a SIRI checklist and an intensive Peer support to hospitalized PWID. Further, we anticipate that the SIRI Checklist and Intensive Peer Intervention will increase use of hospital-based services (HIV, HCV screening, MOUD) and linkage to community-based care: PrEP prescription, MOUD prescription, and related outpatient visit(s).

## Study Objectives and Specific Aims

The overarching objective of this study is to test the feasibility of a SIRI checklist aimed at increasing evidence-based treatment for hospitalized PWID. We will reach this objective through the following AIMS:

Aim 1) Determine the feasibility of a pilot SIRI Checklist and Intensive Peer Intervention for hospitalized PWID to increase uptake of OUD treatment and PrEP and facilitate linkage to community-based services. Feasibility will be measured via recruitment and retention of participants, completion of each portion of the intervention (baseline surveys and follow up assessments), and ability to contact patients post-discharge for intensive Peer support and/or completion of follow up assessments. We will also evaluate the ability to extract documentation of the SIRI Checklist and Peer documentation provider notes.

Aim 2) Quantify risk behaviors in a contemporary Southern cohort of PWID and evaluate Secondary Patient-Centered Outcomes for participants enrolled in the study. We will use validated tools to survey hospitalized PWID on substances used and methods of use, HIV risk behaviors, and substance use stigma. We will also explore the preliminary impact of this low-tech checklist on evidence-based services, such as initiation of buprenorphine, PrEP, HCV screening, and referral to community care. These measures will be compared with treatment groups that do not receive the SIRI Checklist.

## Methods/Design

### Setting

We will recruit 60 participants who are hospitalized for SIRI at the University of Alabama at Birmingham (UAB) Hospital to participate in this pilot clinical trial. The UAB Health system, anchored by the 1157-bed tertiary care center of UAB Hospital, is the largest in the state, and it provides care for PWID referred from other health systems across the Southeast [4].

### Study Design

To evaluate the feasibility of the checklist and intensive peer, we will conduct a pilot randomized clinical trial of the two interventions in which participants will be randomized in a 2x2 factorial design. Process outcomes from this trial, as described in Aim 1, will be used to determine feasibility [15].

#### Aim 1: Primary Feasibility Outcomes:

In Aim 1, we will pilot a SIRI Checklist and Intensive Peer Intervention. We will assess the feasibility of this low-tech checklist and Peer intervention on linkage to evidence-based services, such as initiation buprenorphine, PrEP, and referral to community care. Study feasibility will be defined as the ability to successfully recruit and retain study participants, successful completion of baseline surveys and follow up assessments, ability to contact patients post-discharge for intensive Peer support and completion of follow up assessments, ability to extract documentation of the SIRI checklist, and Peer documentation in provider notes. Participating Addiction and Infectious Disease providers will receive a standardized survey at study completion to elucidate challenges and opportunities to improve the SIRI checklist.

#### Aim 2: Secondary Patient-Centered Outcomes:

In Aim 2, we will use validated tools to survey participants on specific substance use and behaviors, stigma, HIV risk, and preferences around care delivery. Specifically, we will use the 14-item Behavioral Risk Assessment for Infectious Diseases (BRAID) scale, which queries drug use and sexual behaviors while using drugs, and the Substance Use Stigma Mechanisms Scale (SU-SMS), an 18-item validated, theoretically-based assessment [16, 17]. The first component includes surveys that will be administered electronically via tablet in private hospital rooms to reduce social desirability bias (appendix 1) [18]. The second component will be a 3-month follow up survey to assess use of PrEP, addiction treatment, and healthcare utilization. These sessions will last 30 minutes and 45–60 minutes, respectively. Each survey session will include a $30 incentive for patients.

Preliminary treatment outcomes will be explored using hospital data, such as rates of screening for HIV and receipt of HIV prevention (PrEP).

Traditionally, research on PWID is limited by a lack of retention beyond 30 days. To test the feasibility of retaining patients in the study for 3 months, we will work with a professional company, Community Tracking Service (CTS), with expertise in identifying and conducting exit interviews in persons who use drugs as part of extramurally funded research. After three attempts to reach study participants for the exit interview, our study staff will notify our partners at CTS who will then attempt to reach the participant up to one-month post-referral. These attempts may be over phone or in-person at locations identified by participants as part of their intake survey. Open-ended questions will solicit feedback on specific aspects of the checklist or delivery that need to be addressed, edited, or removed before a subsequent randomized controlled study [19]. For participants who do not complete the exit interview, we will review the UAB and coroner data to determine if hospitalized or deceased.

**Figure 1:**
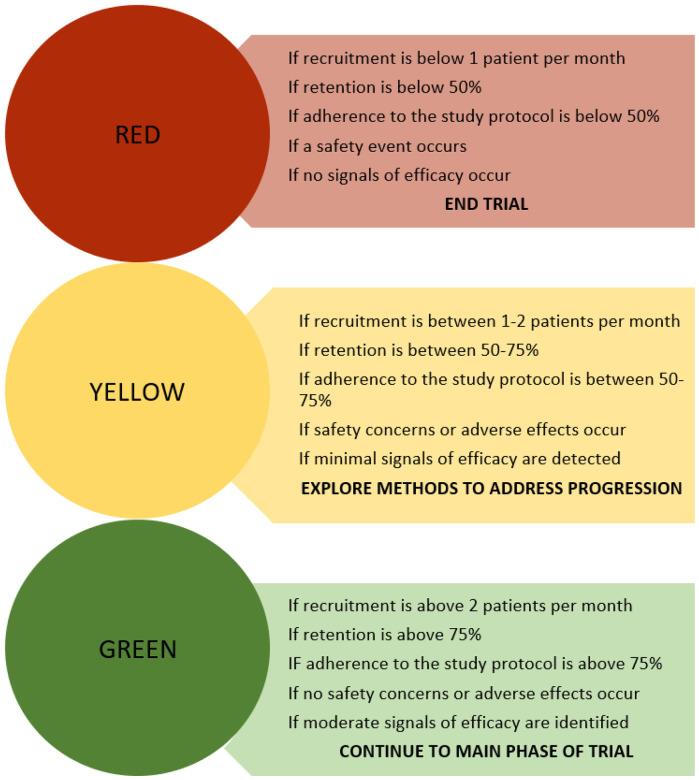
Red, Yellow, Green Progression Criteria

To evaluate the transition to the main trial, we will utilize progression criteria around 1) recruitment and retention, 2) protocol adherence, 3) safety and adverse effects, and 4) signals of efficacy [14]. We will utilize the red, amber, green system for progression criteria [20]. (Fig. 1) For recruitment, we expect to encounter 3 to 4 eligible participants monthly, based on previous studies we have conducted within this population. Given that some of these individuals will refuse to participate in the study, we expect to enroll 2 to 3 participants each month. If we maintain recruitment of 2 or more patients per month, we expect to reach our goal of enrolling 60 participants (15 participants for each arm of the study) within 24 months. If recruitment falls below 2 patients per month, we will explore alternate methods for recruitment to improve recruitment rates. If recruitment stalls and no patients are recruited for more than two consecutive months, the trial will terminate. Retention will be determined by documenting the proportion of: participants who complete weekly calls with their peer, peer calls completed, and participants who complete the follow up survey at 3 months. The target retention rate for each of these rention progression criteria is 75% [21].

To ensure adherence to the study protocol, we will monitor patient documentation to determine whether the ShaPe “smart phrase” was utilized in patient notes. We expect that this smart phrase will be included in all participant documentation after implementation. If adherence to the study protocol falls below 80%, we will revisit the protocol with providers to determine whether further training or modifcations are necessary [21].

The third progression criterion we will employ is participant safety and adverse effects. For this criterion, we will use a Data Safety Monitor model (DSM). A DSM will be designated as an independent faculty member charged with ensuring that the safety of study subjects is protected and that the scientific goals of the study are being met. To support those purposes, the DSM will review any proposed amendments to the study protocol, perform expedited monitoring of all serious adverse events, perform ongoing monitoring of drop-outs and non-serious adverse events, determine whether study procedures should be changed or the study should be halted for reasons related to the safety of study subjects, and perform periodic review of the completeness and validity of data to be used for analysis of safety and efficacy. The DSM will also ensure subject privacy and research data confidentiality.

Finally, to evaluate signals of efficacy, we will evaluate whether participants have received the recommended services that are included in our ShaPe intervention, such as HIV and HCV screening, MOUD prescriptions, and HIV PrEP referrals. Specifically, we will evaluate whether these measures are enacted after the inclusion of the smart phrase text. This data will be collected via provider documentation and the 3-month follow-up survey with participants.

These progression criteria will be used to determine protocol adjustments to be made prior to enacting a larger study. For example, should the recruitment timeline be insufficient, we will evaluate whether the intended population is large enough for a study to be conducted and if so, how we could better identify eligible participants to be enrolled in the study. Adjustments to the smart phrase text will be made to account for under utilization in patient notes and insufficient delivery of recommended services to participants. We will also determine whether the number lost to follow-up reflects adequate usage and efficiency of CTS. In the event that these adjustments are not adequate for a larger study to be conducted, we will establish a new protocol for study.

All amendments and changes to protocol must be approved by UAB IRB before enacted.

### Standard of Care

The standard of care for PWID admitted to UAB Hospital with SIRI includes an Infectious Diseases and Addiction Medicine consultation [4]. Peer support staff meet with all SIRI patients daily during their admission with varying frequency, but peer support does not routinely extend beyond discharge. Over 125 SIRI cases are treated annually: skin, soft tissue, blood, and heart infections. Most have opioid use disorder (OUD), 30% have comorbid methamphetamine use [22, 23]. In a recent survey of 20 PWID at UAB, 50% reported condomless sex and 75% reported sharing injection materials, yet none received PrEP Only 37% were discharged with lifesaving medications for OUD, like buprenorphine [7].

### Intervention

This study will test two interventions. The SIRI Checklist will build on the existing standard of care for SIRI by adding a standardized reminder to offer MOUD, HIV risk assessment, and refer to appropriate community-based care. SIRI Checklist was inspired by the iCARE checklist, developed for endocarditis in PWID, but is applicable for all PWID and will incorporate HIV prevention, harm reduction education, and linkage to care, including HCV treatment [5].

For participants who are randomized to receive the SIRI checklist, providers will receive instructions on how to insert a 5-item checklist into their documentation using a smart phrase text for ease of use and standardization (appendix 2). Because use of a checklist alone has been shown to change provider behavior, providers will not be required to perform any additional tasks other than daily documentation of the checklist. We anticipate that documentation of evidence-based services using a checklist reminder will increase the likelihood that participants will receive them.

The intensive peer intervention will include weekly calls to participants for 4 weeks following discharge. If participants do not respond, peers will make 3 attempts to reach participants at their preferred contact number.

### Recruitment

#### Eligibility Criteria

##### Inclusion Criteria:

We will include 60 PWID with serious injection related infections (SIRI) who are HIV negative, ≥ 18 years old and receiving care at UAB Hospital for each of the three aims.

##### Exclusion Criteria:

We will exclude those unable to provide informed consent due to acute illness or intoxication and those who are living with HIV in order to inform HIV prevention interventions.

#### Sampling and Recruitment Procedures

**Figure 2:**
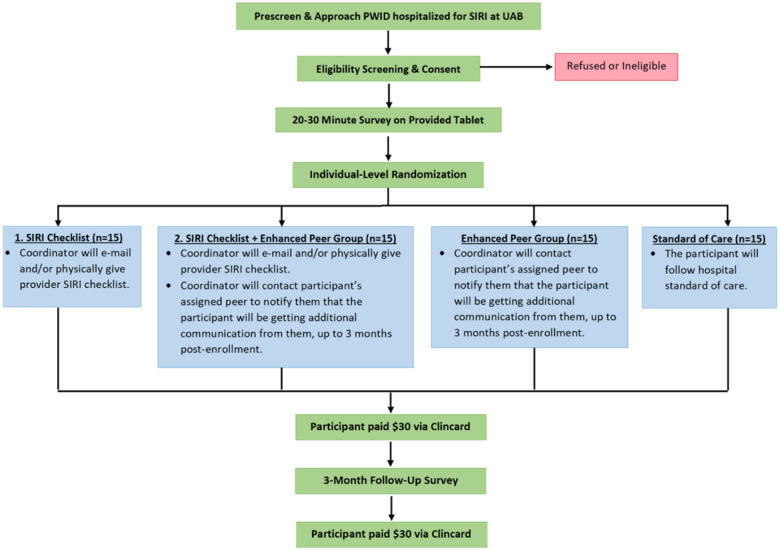
Flow Diagram of ShaPe.

Participants will be recruited following hospitalization for SIRI at UAB Hospital. Our research coordinator will meet biweekly with the Addiction Medicine social worker and/or nurse practitioner, to identify eligible PWID who express interest in study participation. The research team member will then discuss the study with those who are eligible, and if the participant expresses interest, the informed consent process will be completed (appendix 3). We will provide phones for participants who do not have access to a phone. The participant will then complete the survey used to accomplish Aim 1. Following survey completion, the participant will be randomly sorted using a 2x2 Factorial Design, into one of the four groups: SIRI Checklist, SIRI Checklist + Enhanced Peer, Enhanced Peer, and Standard of Care. Randomization will be performed in advance using blocks of n = 4 and n = 8. A total of 60 participants will be recruited, 15 per group, which is in alignment with sample size guidance for pilot and feasibility studies [24]. For incentive, patients will receive a $30 gift card at enrollment, as well as a $30 gift card at the 3-month follow up (Fig. 2).

### Data Sources

We will collect data on stigma, HIV awareness and risk behaviors via electronic surveys; results will be stored in a RedCap database [25]. We will obtain data on sociodemographics and clinical factors, like type of infection and HIV and HCV test results, using data extracted from the Electronic Medical Record (EMR) and managed in a RedCap database. Prescription data and healthcare utilization data will also be obtained from the EMR. We will extract outcomes related to linkage to prescription for PrEP and/or MOUD on discharge, and attendance at provider visit(s) related to HIV and/or addiction services following hospitalization. Data extraction will be limited to services provided in the UAB health system.

Participating providers who serve PWID will be asked to complete a standardized survey of the study and intervention in order to determine the study feasibility and identify opportunities to improve.

### Analysis

To evaluate our primary aim, feasibility will be evaluated through time and number of eligible participants required for participant recruitment, participant retention, and completion rates. Acceptability will be evaluated through patient enrollment and provider survey responses towards the SIRI checklist and Intensive Peer.

As a secondary outcome, we will explore the participant data and summarize sociodemographic and clinical factors of participants using descriptive statistics (measures of central tendency, dispersion, and distribution) and confidence intervals. Clinical factors will include initiation of buprenorphine, PrEP, HIV and HCV screening, and referral to community care. Lastly, we will evaluate the provider survey data to identify overall trends in provider responses.

These results are planned for presentation at national conferences and inclusion in manuscripts for publication in high impact journals.

## Discussion

Results from this study will enable further research aimed at designing and testing patient-centered interventions towards evidence-based care for PWID. For example, preliminary survey data on risk behaviors and stigma will provide opportune insight into approaching addiction and infectious disease care for this patient population. Additionally, the use of the Community Tracking Service could expand current research opportunities with PWID, a population that is not often retained for extended time of study. Increased retention time would allow for the expansion of multiple projects revolving post-discharge survey data. Finally, feasibility of this pilot study would permit further usage and study of the SIRI Checklist and Enhanced Peer intervention in a larger population. Developing a framework for the uptake of HIV prevention, HCV services, and addiction treatment for hospitalized patients is essential to reach patients with limited access to community care due to poverty, rurality, and resource poor communities in the Deep South.

## Figures and Tables

**Table 1.** Study Timeline for ShaPe.

	Enrollment	Allocation	Post- allocation	Close-out
TIMEPOINT**	*Baseline*	0	*3-month* *follow-up*	*3 months* *post-* *enrollment*
**ENROLLMENT:**				
***Eligibility screen***	X			
***Informed consent***	X			
***Baseline Survey***	X			
***Allocation***		X		
**INTERVENTIONS:**				
***SIRI Checklist***	X			
***SIRI Checklist + Enhanced Peer***	X			
***Enhanced Peer***	X			
***Standard of Care***	X			
**ASSESSMENTS:**				
***Baseline Variables***				
*Demographics*	X			
*Medications for Opioid Use Disorder (MOUD) use at admission*	X			
*PrEP use at admission*	X			
***Secondary Outcome variables (HIV, HCV testing, MOUD, PrEP)***				X
*Feasibility*				X
*Acceptability*				X
***Secondary Outcome variables***				
*Number of prescriptions for PrEP*			X	
*Number of prescriptions for medications for MOUD*			X	
*Number of outpatient visits attended related to HIV &/or addiction services following hospitalization.*			X	

## Abbreviations

HIV: human immunodeficiency virus
HCV: hepatitis C virus
PWID: persons who inject drugs
PrEP: pre-exposure prophylaxis
SIRI: serious injection related infections
OUD: opioid use disorder
MOUD: medication for opioid use disorder
UAB: University of Alabama at Birmingham
CTS: community tracking service
EMR: electronic medical record
DSM: Data Safety Monitor
